# Sexually dimorphic effects of oxytocin receptor gene (*OXTR *) variants on Harm Avoidance

**DOI:** 10.1186/2042-6410-3-17

**Published:** 2012-07-30

**Authors:** Trayana Stankova, Peter Eichhammer, Berthold Langguth, Philipp G Sand

**Affiliations:** 1 Experimental and Clinical Neurosciences Graduate Program, University of Regensburg, Regensburg, Germany; 2 Department of Psychiatry and Psychotherapy, University of Regensburg, Universitaetsstrasse 84, 93053 Regensburg, Germany

**Keywords:** Oxytocin receptor gene, Personality traits, Sexual dimorphism

## Abstract

**Background:**

Recent research has suggested that oxytocin receptor gene (*OXTR*) variants may account for individual differences in social behavior, the effects of stress and parenting styles. Little is known, however, on a putative role of the gene in heritable temperamental traits.

**Methods:**

We addressed effects of two common *OXTR* variants, rs237900 and rs237902, on personality dimensions in 99 healthy subjects using the Temperament and Character Inventory.

**Results:**

When sex was controlled for and an *OXTR* genotype*sex interaction term was included in the regression model, 11% of the variance in Harm Avoidance could be explained (uncorrected p ≤ 0.01). Female carriers of the minor alleles scored highest, and a novel A217T mutation emerged in the most harm avoidant male participant.

**Conclusions:**

Findings lend support to a modulatory effect of common *OXTR* variants on Harm Avoidance in healthy caucasian women and invite resequencing of the gene in anxiety phenotypes to identify more explanatory functional variation.

## Background

The neuropeptide oxytocin is synthesized in magnocellular neurons of the hypothalamus and is released into the blood stream from the posterior pituitary stimulating smooth muscle contractions during labor and birth. G-protein-coupled oxytocin receptors (OXTR), however, mediate not only oxytocin peripheral actions in reproduction but also a range of central nervous actions that regulate human social behavior [1]. From investigations of the gene encoding OXTR on human chromosome 3p26, associations have emerged with disrupted affialitive behavior [2], the seeking of emotional support [3], and attachment anxiety in women [4], among other traits. At the brain structural level, variation in the *OXTR* gene would appear to affect amygdala volume [5], indicating a possible vulnerability to the adverse effects of stress [6]. Functional imaging experiments strongly suggest that stress-induced dopamine release and increased attachment anxiety are both under control of *OXTR* variation [7]. In animals, patterns of OXTR expression in the central nervous system have long suggested a contribution of OXTR to the regulation of anxiety [8] and to defensive maternal behavior [9].

Although the spectrum of *OXTR*-related candidate phenotypes has thus greatly expanded, it is still unclear which personality traits should be considered a part of this spectrum. Animal models have implicated oxytocin effects in two temperaments, Novelty Seeking (NS) and Harm Avoidance (HA) [10], but a study of human *OXTR* variants has pointed to a third temperamental factor, Reward Dependence (RD) [11]. Most recently, a role has been proposed for *OXTR* variation in predicting optimism and self-esteem [12,13], i.e. traits negatively correlated with HA [14]. According to Cloninger’s psychobiological model of temperament and character, NS is related to behavioral activation, HA to behavioral inhibition or anxiety-proneness, and RD to behavioral maintenance. In order to shed more light on this issue, we readdressed the impact of *OXTR* variation on personality dimensions using the Temperament and Character Inventory (TCI) [15].

## Materials and methods

106 healthy unrelated Caucasian subjects were recruited among hospital staff and their friends in the Regensburg area. All provided informed consent, donated a blood sample, and were administered the 226-item German Temperament and Character Inventory (TCI) version 8 [15]. The TCI evaluates four higher order temperament and three higher order character traits. Each of the seven traits is multifaceted, i.e. it consists of lower order components. On the whole, the TCI is made up of twenty- five facets (12 facets of temperament and 13 facets of character) of which we examined only the genetically independent main temperamental factors, Novelty Seeking, Harm Avoidance, Reward Dependence, and Persistence [16]. After excluding incomplete questionnaires, 99 volunteers (48 men and 51 women, mean age 29.1 ± 7.4 years and 28.7 ± 7.7 years, respectively) were genotyped for two *OXTR* variants, rs237900 and rs237902 by Sanger sequencing (Primers: 5‘-GCAGGTGCACATCTTCTCTC-3‘, forward, and 5‘-GGAGTCCCTTGAACCTGTTT-3‘, reverse). The variants were selected for having previously been implicated in Novelty Seeking (rs237902, see [17]), for a heterozygosity of >0.4 (both variants), and for their inclusion in major commercial SNP arrays (rs237900). Sequence alignments were conducted with DNA Dynamo 1.0 (Blue Tractor Software, UK). STATA 8.0 (Stata Corporation, College Station, TX, USA) was used for descriptive statistics and multiple regression. The level of statistical significance was set at p < 0.05. Evolutionary conservation in 46 mammals was assessed with a phylogenetic hidden Markov model-based method, phastCons [18]. A probabilistic classifier incorporating secondary structure information (PolyPhen2) was used to refine this estimate for nonsynonymous *OXTR* variants.

## Results

**Figure 1:**
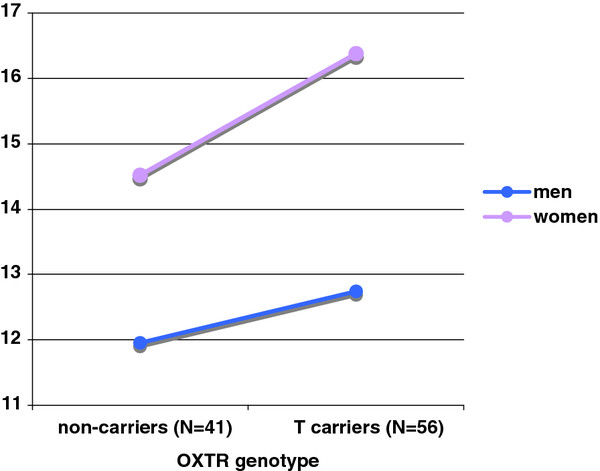
**Interaction of *OXTR *genotype ( *t *=1.26, *p *=0.20) and sex ( *t *=−3.05, *p *=0.003) as predictors of Harm Avoidance mean scores.**

TCI scores conformed to a normal distribution for NS, HA, and RD, but not for PS. Consistent with previous research [19], PS scores were therefore log-transformed before statistical analyses to ensure Gaussian distributions across all scales (Shapiro-Wilk test of transformed data *p* > 0.67; reported means and SDs are not transformed for ease of interpretation). Effects of rs237900 and rs237902 on the TCI temperamental scales were non-significant when data from men and women were pooled (*F* < 1.25, *p* > 0.26). However, when sex was controlled for, both *OXTR* variants under study predicted Harm Avoidance in multiple regression models (*p* = 0.01 for rs237902, *p* = 0.006 for rs237900). Considering moderate to strong intermarker linkage disequilibrium (*r*^*2*^ = 0.75), we limited our analyses to rs237900 (Table 1). To avoid trade-offs in power, CT and TT genotypes were collapsed into T carriers and were then contrasted with the remaining subjects under the assumption of a recessive mode of inheritance (mean HA scores ± SD: CC = 13.4 ±4.7, CT = 14.3 ±6.3, TT = 14.3 ±5.1). Following a Bonferroni correction for eight separate regressions (effects of genotype on four temperamental scales with and without sex as a second factor), significance was still achieved (*p* = 0.045). Female carriers of the minor allele (T) scored higher on Harm Avoidance than did all other subjects. Even though only sex appeared to exert a main effect in the additive model (Figure 1), the variance explained by both regressor variables reached 11% when an interaction term was included (Table 1, 10% assuming no interaction). No noteworthy effect was observed on Novelty Seeking, Reward Dependence or Persistence. 

**Table 1:** **TCI temperament scores (mean ±SD) for 99 men and women, stratified by*OXTR*genotype**

***OXTR*genotype**^**a**^	**NS**	**HA**	**RD**	**PS**
male T-allele carriers (*N*=27)	20.0 ±6.1	12.7 ±5.4	15.0 ±3.7	4.4 ±2.1
female T-allele carriers (*N*=29)	22.3 ±6.1	16.4 ±5.4	16.5 ±2.9	3.4 ±1.9
male non-carriers (*N*=20)	21.5 ±6.1	11.9 ±4.5	16.5 ±3.6	4.1 ±1.8
female non-carriers (*N*=21)	22.8 ±4.4	14.5 ±5.0	15.4 ±3.2	4.0 ±1.9
effects of predictor variables				
*F* (2,94) _[sex rs237900]_	1.615	5.460	0.215	1.396
*p*_[sex rs237900]_	0.204	0.005	0.807	0.253
*p*_[corrected]_	1.000	0.046	1.000	1.000
*F* (3,93) _[including sex*rs237900]_	1.144	3.693	1.407	1.327
*p*_[including sex*rs237900]_	0.335	0.014	0.246	0.270

When sex is controlled for and a recessive mode of inheritance is assumed, rs237900 predicts Harm Avoidance in additive and multiplicative regression models.

significance tests refer to log-transformed TCI scores.

^a^ results are shown for rs237900, 2 unreadable ABI trace files.

NS=Novelty Seeking, HA=Harm Avoidance, RD=Reward Dependence, PS=Persistence.

**Figure 2:**
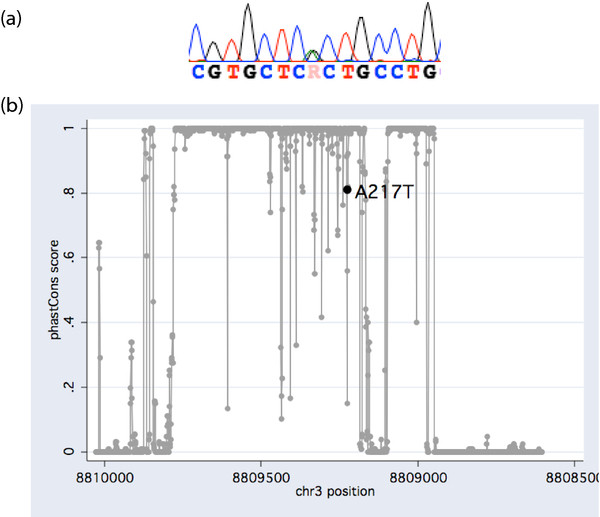
**Chromatogram (a) and evolutionary conservation plot (b) of a newly identified *OXTR *mutation, A217T, in a subject scoring on the 98th percentile for Harm Avoidance. **Basewise conservation of the amplicon sequence is plotted from 5‘ to 3‘ against the physical position on chromosome 3 using 46 placental mammals featured in the UCSC Genome Browser. A217T maps to a highly conserved region encoding the 5th OXTR transmembrane domain.

Detailed examination of the sequence chromatograms revealed one carrier of a novel synonymous variant, Y200Y (TAC > TAT), plus one carrier of a novel missense mutation, A217T (GCT > ACT). The latter coincided with the highest Harm Avoidance score of all male subjects, and the 98th percentile of pooled Harm Avoidance scores under a Gaussian distribution. To obtain more detailed information on the prevalence of this substitution in the general population, we screened an additional set of previously recruited control subjects (healthy by self-report, no TCI data available) originating from the greater Regensburg area using the same experimental protocol (*N* = 396, 172 men and 224 women). They comprised university students, other hospital employees, and their acquaintances. No further carriers were identified, lowering the total allele frequency to 0.001. A217T maps to a region encoding the fifth OXTR transmembrane domain (TMD), delimited by residues 203 and 225. Evolutionary conservation of this region in mammals is high (Figure 2) and *in silico* predictions indicated that the variant is possibly damaging (PolyPhen2 score 0.46). Two known *OXTR* variants, rs4686302, rs61740241, were confirmed in the course of screening the extension sample, plus two novel intronic variants (T > C, chr3: 8,808,677 and G > A, chr3: 8,808,925). Two subjects also carried one novel *OXTR* missense variant each, E242K (GAG > AAG) and G252A (GGG > GCG) that, however, do not map to any TMD and appear benign (PolyPhen2 score <0.01).

## Discussion

The present results extend previous work on *OXTR* sequence variants’ role in predicting facets of human behavior. Under the assumption of sexually dimorphic effects, an association was observed for two substitutions and Harm Avoidance, with female carriers of the minor alleles scoring highest.

The possible implications of this observation are three-fold:

From an evolutionary perspective, oxytocin control of Harm Avoidance in humans is relevant to parental care and related behaviors. According to Cloninger, harm avoidant reaction patterns have arisen from multifactorial phylogenetic mechanisms which in their 'optima' are advantageous to the adaptation and survival of the individual [20]. Human offspring require more intense care and protection from hazards in early life than other species. Both the fine-tuning of appraisal mechanisms that detect potential threats to the offspring and the initiation of alarm responses in parents are key to averting hazards. There is a growing concern, however, that high levels of anxiety or excessive harm avoidant behavior in parents may be counter-productive and, in some age-groups, may even augment childrens' levels of anxiety [21]. Oxytocin exerts anxiolytic effects that can balance and optimize maternal defense. Consistent with this view, breast-feeding women show lower systolic blood pressure reactivity to aggressive encounters than do formula-feeding women [22]. Similar observations have been made during lactation using cold pressor and mental arithmetic stressor tasks [23]. Provided that OXTR are involved in these functionalities, and provided the present association is genuine, women carrying the minor *OXTR* alleles could be less susceptible to the anxiety-attenuating effects of oxytocin than subjects carrying only wild-type alleles and, possibly, more prone to responding aggressively to detected threats. While a certain measure of heightened reactivity to stressful stimuli should benefit the defense of offspring, excessive emotional lability is liable to interfere with sensitive parenting [24]. Sex-specific responses to oxytocin could be one reason why HA is gender-sensitive [25]. Until this is confirmed, the absence of a main effect of genotype on HA scores in men cautions against overrating the predictive role of *OXTR* in shaping personality traits.

From a more general point of view, the gene encoding OXTR has been implicated in the responsiveness to social support as a buffer against stress [26]. This extends the reciprocal interaction between serotonin and social behavior to the oxytocinergic system [24,27]. To specifically evaluate gene x environment interactions of the SNPs examined, however, more work is needed in a context of psychosocial stress exposure. Theoretically, a putative loss of OXTR function could be mediated either by the T alleles, or by mutations that are in linkage disequilibrium with these alleles. Of the two variants addressed here, rs237900 is intronic, and its functionality is uncertain. Rs237902 stands for a synonymous substitution at residue 230 and has the potential to interfere with gene expression, e.g. by disrupting motifs recognized by transcription factors, or by affecting post-transcriptional regulatory mechanisms. Confirmatory investigations in larger samples are still required, together with an elucidation of the mechanism by which the endocrine response is altered.

In contrast to the results described earlier for rs53576 [11], we failed to identify significant genetic effects on the personality dimension of Reward Dependence. This could be due to a difference in sample size, or to using different markers. Others have shown that rs237902 and rs53576 map to two different haplotype blocks in the Caucasian population [2]. Balancing of Harm Avoidance and Reward Dependence would appear to facilitate response selection mechanisms in parental care. On the one hand, sources of reward trigger heightened distress when the attachment object is being threatened. Thus reward-related reactivity of mothers to images of infants (as measured by activation of the ventral striatum) varies with cues of potential harm [28,29]. On the other hand, reward responses are believed to buffer otherwise deleterious levels of heightened anxiety and stress [30]. On these grounds, common *OXTR* variants could serve to predict Harm Avoidance and Reward Dependence, depending on the markers used, and on the assumptions made in the respective models. It is noteworthy that sexually dimorphic effects of *OXTR* variants are emerging in the sensitivity to pain [7], a composite trait integrating, in turn, measures of Harm Avoidance and Reward Dependence [31].

Finally, identification of a novel A217T mutation in a highly harm avoidant subject may be a chance finding. Whether a putative loss of receptor function is aggravated any further by this rare substitution is unresolved. Its location in a highly conserved region argues against a neutral effect on the OXTR protein and should therefore justify inclusion in future investigations of target phenotypes.

## Summary and conclusion

The experiments discussed here are the first to rationalize a role of the oxytocin receptor gene in Harm Avoidance and incentivize resequencing the *OXTR* gene in conditions marked by an increased sensitivity to perceived threat. We acknowledge that extrapolations of our findings to oxytocin-mediated adaptive responses in the context of parenting, or to stress responses in general, are, at this stage, largely tentative. *OXTR* variants may predispose to developing less specific behavioral symptoms by interfering with visual attention and awareness [32]. Moreover, most investigations of parenting have focused on maternal behavior. More detailed assessments are now warranted to replicate the association of *OXTR* with HA, adding to the emerging association of *OXTR* variants with parenting styles in mothers and fathers [33].

## Competing interests

The authors declare that they have no competing interests.

## Authors' contributions

PGS designed the study and wrote the protocol. TS conducted the experiments and assisted PGS with statistical analysis. PE supervised the recruitment of subjects who took part in the study. BL and TS managed the literature searches, analyzed the data and helped to draft the manuscript. All authors contributed to and have approved the final manuscript.
